# Novel local learning rule for neural adaptation fits Hopfield memory networks efficiently and optimally

**DOI:** 10.1186/1471-2202-14-S1-P215

**Published:** 2013-07-08

**Authors:** Chris Hillar, Jascha Sohl-Dickstein, Kilian Koepsell

**Affiliations:** 1 Redwood Center for Theoretical Neuroscience, Berkeley, CA 94720, USA

## 

We present an algorithm to store binary memories in a Little-Hopfield neural network using minimum probability flow, a recent technique to fit parameters in energy-based probabilistic models. For memories without noise, our algorithm provably achieves optimal pattern storage and outperforms classical methods both in speed and memory recovery. Moreover, when trained on noisy or corrupted versions of a fixed set of binary patterns, our algorithm finds networks which correctly store the originals. We also demonstrate this finding visually with the unsupervised storage and clean-up of large binary fingerprint images from significantly corrupted samples.

## Background

In 1982, motivated by neural modeling work of [3] and the Ising spin glass model from statistical physics [2], Hopfield introduced a method for the storage and retrieval of binary patterns in an auto-associative neural-network [1]. However, existing techniques for training Little-Hopfield networks suffer either from limited pattern capacity or excessive training time, and they exhibit poor performance when trained on unlabeled, corrupted memories.

## Results

Our main theoretical contributions here are the introduction of a tractable and neurally-plausible algorithm MPF for the optimal storage of patterns in a Little-Hopfield network, a proof that the capacity of such a network is at least one pattern per neuron, and a novel local learning rule for training neural networks. Our approach is inspired by minimum probability flow [4], which fits probabilistic models without computations with a partition function.

**Figure 1:**
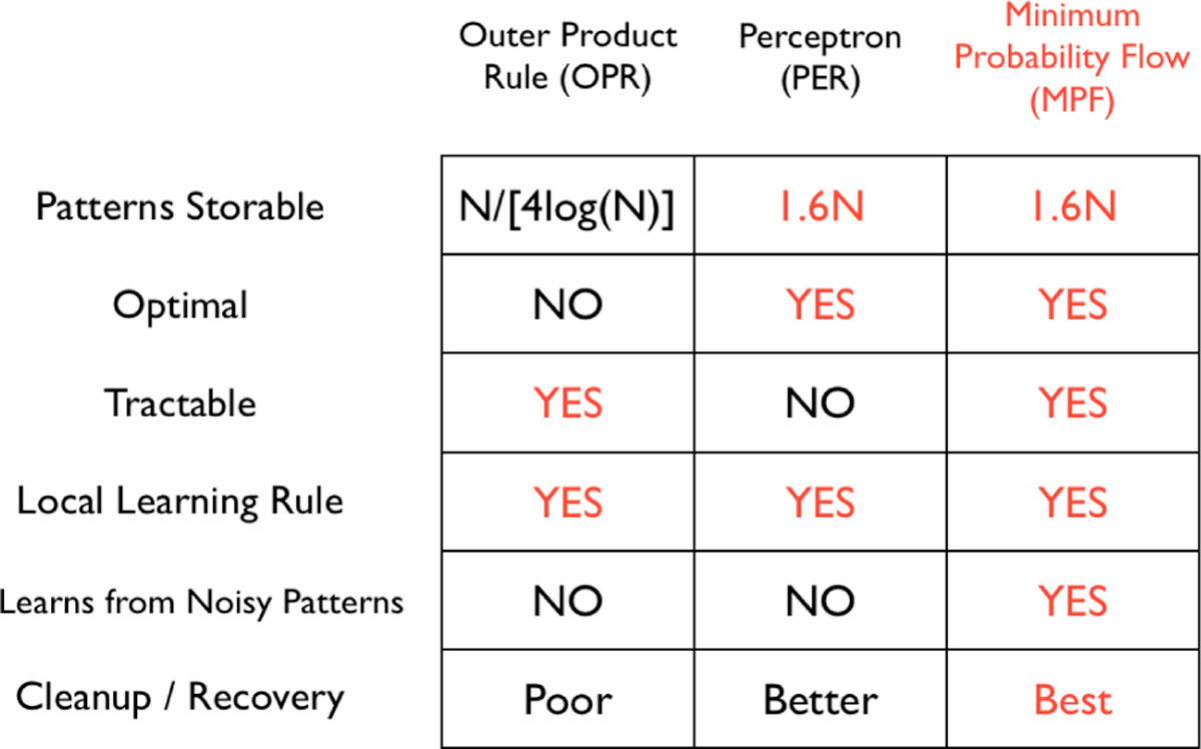
**Performance**. Comparison of our training algorithm (MPF) to classical methods Outer Product Rule (OPR) and Perceptron (PER).

**Figure 2:**
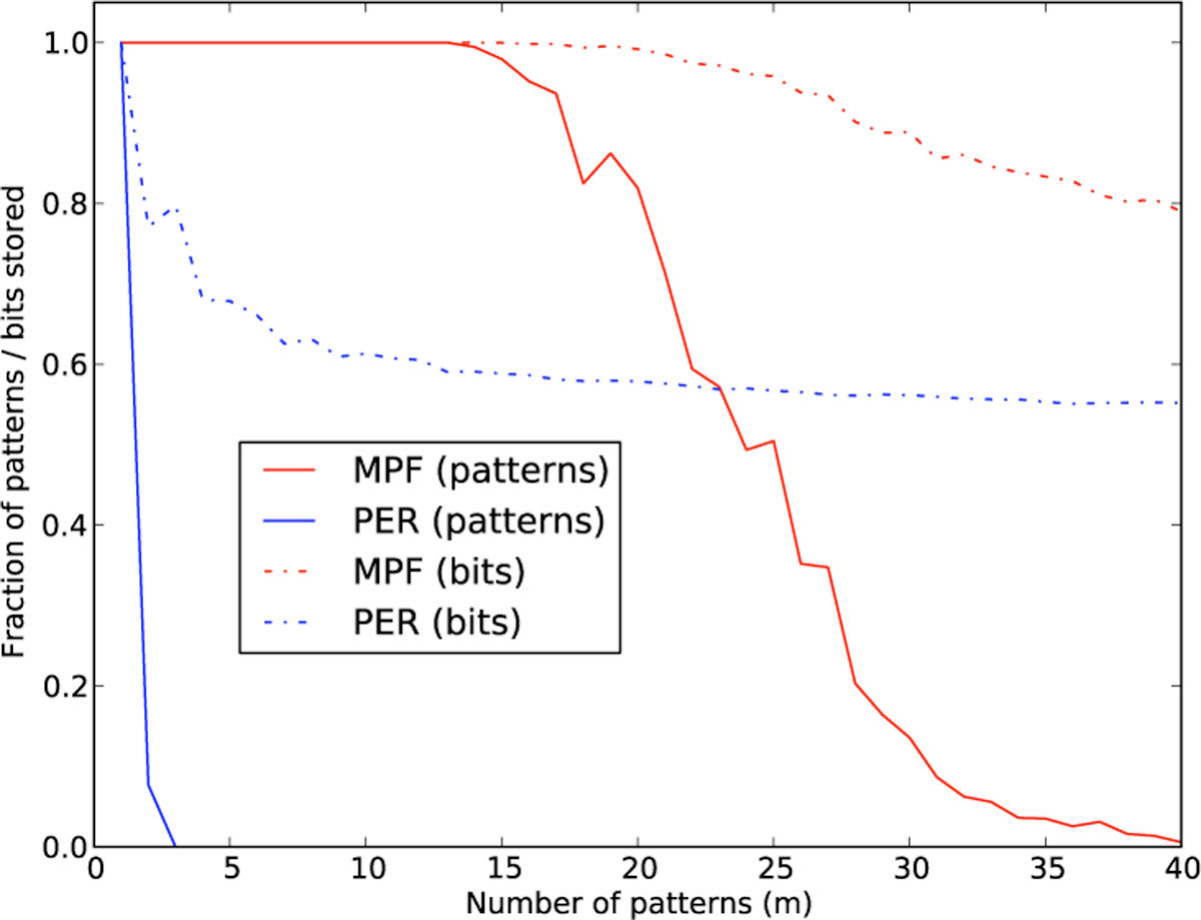
**Noisy data storage**. Shows fraction of patterns (red for MPF, blue for PER) and fraction of bits (dotted red for MPF, dotted blue for PER) recalled of trained networks (*n *= 64 nodes each) as a function of the number of patterns *m *to be stored. Training patterns were presented repeatedly with 20 bit corruption (i.e., 31% of the bits flipped).

We also present several experimental results. Compared with standard techniques for Little-Hopfield pattern storage, our method is shown to be superior in efficiency and generalization (Figure 1). Another finding is that our algorithm can store many patterns in a Little-Hopfield network from highly corrupted (unlabeled) samples of them (Figure 2). We also store 64 × 64 binary images of human fingerprints from highly corrupted versions (Figure not shown).
